# Rheumatoid arthritis response to treatment across IgG1 allotype – anti-TNF incompatibility: a case-only study

**DOI:** 10.1186/s13075-015-0571-z

**Published:** 2015-03-18

**Authors:** Ariana Montes, Eva Perez-Pampin, Federico Navarro-Sarabia, Virginia Moreira, Arturo Rodríguez de la Serna, Berta Magallares, Yiannis Vasilopoulos, Theologia Sarafidou, Antonio Fernández-Nebro, María del Carmen Ordóñez, Javier Narváez, Juan D Cañete, Ana Marquez, Dora Pascual-Salcedo, Beatriz Joven, Patricia Carreira, Manuel J Moreno-Ramos, Rafael Caliz, Miguel Angel Ferrer, Rosa Garcia-Portales, Francisco J Blanco, Cesar Magro, Enrique Raya, Lara Valor, Juan J Alegre-Sancho, Alejandro Balsa, Javier Martin, Darren Plant, John Isaacs, Ann W Morgan, Anne Barton, Anthony G Wilson, on behalf of Biologics in Rheumatoid Arthritis Genetics and Genomics Study Syndicate (BRAGGSS), Juan J Gómez-Reino, Antonio Gonzalez

**Affiliations:** Laboratorio de Investigacion 10 and Rheumatology Unit, Instituto de Investigacion Sanitaria - Hospital Clinico Universitario de Santiago, Santiago de Compostela, Spain; Rheumatology Unit, Hospital Universitario Virgen Macarena, Sevilla, Spain; Rheumatology Unit, Hospital Santa Creu e San Pau, Barcelona, Spain; Department of Biochemistry and Biotechnology, University of Thessaly, Larissa, Greece; Servicio de Reumatología, HRU Carlos Haya, Universidad de Málaga, Instituto de Investigación Biomédica de Málaga (IBIMA), Málaga, Spain; Department of Rheumatology, Hospital Universitario de Bellvitge, Barcelona, Spain; Rheumatology Unit, Hospital Clinic, Barcelona, Spain; Instituto de Parasitología y Biomedicina López-Neyra, CSIC, Granada, Spain; Department of Immunology, Instituto de Investigación Hospital Universitario La Paz, Hospital La Paz, Madrid, Spain; Department of Rheumatology, Hospital 12 de Octubre, Madrid, Spain; Department of Rheumatology, Hospital Virgen de la Arrixaca, Murcia, Spain; Rheumatology Unit, Hospital Universitario Virgen de las Nieves, Granada, Spain; Department of Rheumatology, Hospital Virgen de la Victoria, Málaga, Spain; Rheumatology Department, Instituto de Investigacion Biomedica–Complejo Hospitalario Universitario A Coruna, A Coruna, Spain; Department of Medicine, University of Santiago de Compostela, Santiago de Compostela, Spain; Department of Rheumatology, Hospital Clínico San Cecilio, Granada, Spain; Rheumatology Unit, Hospital General Universitario Gregorio Marañón, Madrid, Spain; Department of Rheumatology, Hospital Doctor Peset, Valencia, Spain; Department of Rheumatology, Instituto de Investigación Hospital Universitario La Paz, Hospital Universitario La Paz, Madrid, Spain; NIHR Manchester Musculoskeletal Biomedical Research Unit, Central Manchester University Hospitals NHS Foundation Trust, Manchester Academic Health Science Centre, Manchester, UK; Musculoskeletal Research Group, Institute of Cellular Medicine, The Medical School, Newcastle University, Newcastle, UK; National Institute for Health Research Newcastle Biomedical Research Centre, Newcastle upon Tyne Hospitals NHS Foundation Trust and Newcastle University, Newcastle upon Tyne, Newcastle UK; Leeds Institute of Rheumatic and Musculoskeletal Medicine, St. James’s University Hospital, University of Leeds, Leeds, UK; NIHR Leeds Musculoskeletal Biomedical Research Unit, Leeds Teaching Hospitals NHS Trust, Leeds, UK; Arthritis Research UK-Centre for Genetics and Genomics, The University of Manchester, Manchester, UK; University College Dublin, Dublin, Ireland; Laboratorio Investigacion 10, Hospital Clinico Universitario de Santiago, Edificio de consultas, planta −2 Travesia de Choupana, sn, Santiago de Compostela, 15706 Spain

## Abstract

**Introduction:**

We have hypothesized that incompatibility between the G1m genotype of the patient and the G1m1 and G1m17 allotypes carried by infliximab (INX) and adalimumab (ADM) could decrease the efficacy of these anti-tumor necrosis factor (anti-TNF) antibodies in the treatment of rheumatoid arthritis (RA).

**Methods:**

The G1m genotypes were analyzed in three collections of patients with RA totaling 1037 subjects. The first, used for discovery, comprised 215 Spanish patients. The second and third were successively used for replication. They included 429 British and Greek patients and 393 Spanish and British patients, respectively. Two outcomes were considered: change in the Disease Activity Score in 28 joint (ΔDAS28) and the European League Against Rheumatism (EULAR) response criteria.

**Results:**

An association between less response to INX and incompatibility of the G1m1,17 allotype was found in the discovery collection at 6 months of treatment (*P* = 0.03). This association was confirmed in the replications (*P* = 0.02 and 0.08, respectively) leading to a global association (*P* = 0.001) that involved a mean difference in ΔDAS28 of 0.4 units between compatible and incompatible patients (2.3 ± 1.5 in compatible patients *vs.* 1.9 ± 1.5 in incompatible patients) and an increase in responders and decrease in non-responders according to the EULAR criteria (*P* = 0.03). A similar association was suggested for patients treated with ADM in the discovery collection, but it was not supported by replication.

**Conclusions:**

Our results suggest that G1m1,17 allotypes are associated with response to INX and could aid improved therapeutic targeting in RA.

**Electronic supplementary material:**

The online version of this article (doi:10.1186/s13075-015-0571-z) contains supplementary material, which is available to authorized users.

## Introduction

Advances in the treatment of rheumatoid arthritis (RA) including anti-tumor necrosis factor (anti-TNF) monoclonal antibodies have led to successful control of the disease in many patients [1]. However, it is still necessary to change the initial drug because of poor efficacy in a significant fraction of them. This trial and error approach increases the burden of RA and could consume the early months after RA onset when a better long-term prognosis could be obtained [2]. Confronted with this problem rheumatologists have sought predictive biomarkers to orient drug choice [3,4]. A few have already been found as autoantibody seronegativity that identifies patients with poor response to rituximab, but more are needed [3-6].

An area of recent progress has been awareness of the importance of the blood levels of the biologics at the trough between two treatment doses and of anti-drug antibodies [7-13]. These antibodies could work in two ways to decrease the drug’s efficacy: neutralizing the biologic and increasing its clearance. They are present in most patients showing infusion reactions to infliximab (INX) [12,14,15].

**Figure 1:**
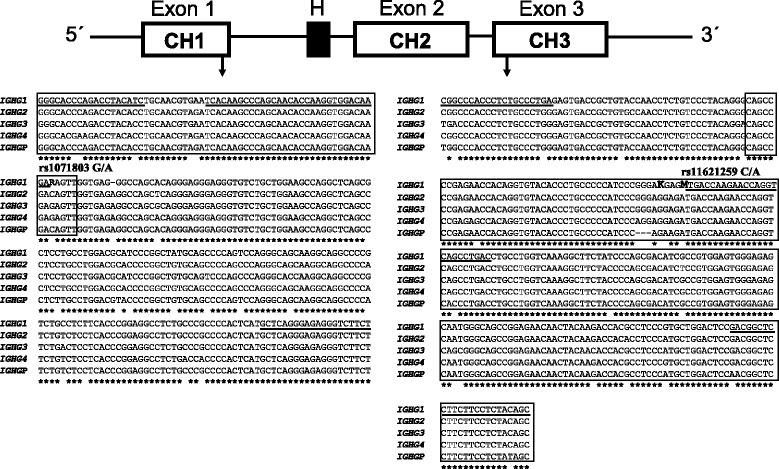
**Genomic structure and sequence of the G1m allotypes.** In the upper graph, the schematic structure of the immunoglobulin heavy constant gamma 1 (*IGHG1*) gene in chromosome14 is shown with white rectangles for the CH exons and a black rectangle for the hinge (H) sequence. Below, the *IGHG1* sequences that were amplified to genotype rs1071803 (for G1m17/G1m3) and rs11621259 (for G1m1/nullG1m1) are shown with the aligned sequences that could be co-amplified with the same primers according to Blastn (megablast, default settings). *IGHG3*, *IGHG2* and *IGHG4* code for the heavy chains of IgG3, IgG2 and IgG4, respectively, while *IGHGP* is a pseudogene. Exon sequences are framed in boxes. The three nsSNPs encoding the allotypes are in bold following the International Union of Pure and Applied Chemistry nomenclature: R for the G/A alleles at *rs1071803*; M for the C/A alleles at *rs11621259*; and K for the G/T alleles at the other nsSNP encoding the G1m1/nullG1m1 allotype. Primers and minisequencing probes are underlined. nsSNP, non synonymous single nucleotide polymorphisms.

A related area of concern for biologics that bear the fragment crystallizable (Fc) of immunoglobulin G (IgG) is the possibility of inducing anti-allotype antibodies or T cell reactions in incompatible patients [16-20]. The allotypes are protein polymorphisms in the IgGs that are able to induce antibodies when injected in incompatible subjects (those not bearing them). There are several human immunoglobulin allotypes, but for the biologics used to treat RA the most relevant are in the heavy chain of IgG1 (Figure 1). INX and adalimumab (ADM), two of the most commonly used anti-TNF monoclonal antibodies, bear the G1m1 and G1m17 allotypes [16,17]. At a genetic level, these two allotypes are in perfect linkage disequilibrium (LD) in Europeans (r^2^ = 1) and, therefore, are referred as the G1m1,17 allotype but the anti-allotype antibodies are directed either against the G1m1 epitope or against the G1m17 epitope [21]. These allotypes are missing in a large fraction of Europeans, who are susceptible to mounting an anti-allotype response when exposed to INX or ADM. Detection of the anti-allotype antibodies targeting biologics has been rare, but this could be due to the technical challenges presented by these antibodies [16,17,19,22]. In addition, exposition to the G1m1 allotype in incompatible subjects induces T-cell responses directed against a different peptide in the IgG1 molecule [18]. The G1m1,17 allotypes could also influence treatment response by modifying the strength of antibody immune responses, as has been shown for several antigens, for rheumatoid factor (RF), and for anti-ADM antibodies [20,23-25]. These differences could be mediated by the allotypes themselves or by other variants in the immunoglobulin heavy chain locus (*IGH*) through LD with the allotypes [26,27].

The *IGH* locus displays a high level of structural complexity, with highly homologous genes (Figure 1) arising from multiple segmental duplication events [26,27]. Consequently, this locus is not adequately covered on genome-wide SNP chips [23,28]. Specifically, none of the SNPs coding for G1m allotypes or their tagging SNPs was included in the genome-wide association studies (GWAS) analyzing the response of patients with RA to treatment with anti-TNF drugs [29-32].

Here, we show that incompatibility at the G1m allotype was associated with less response to treatment with INX. This association was supported by three sets of patient samples, one used for discovery and the other two for replication. The difference between G1m compatible and incompatible patients was small, but of possible utility because it is similar to that observed between seronegative and seropositive patients in the response to rituximab, which is used in clinical practice, and because it was stronger in some patient subgroups. A similar association was found for the patients treated with ADM in the discovery set, but it was not reproduced in the first replication set, which included a considerably larger number of ADM treated patients.

## Methods

### Patients

**Figure 2:**
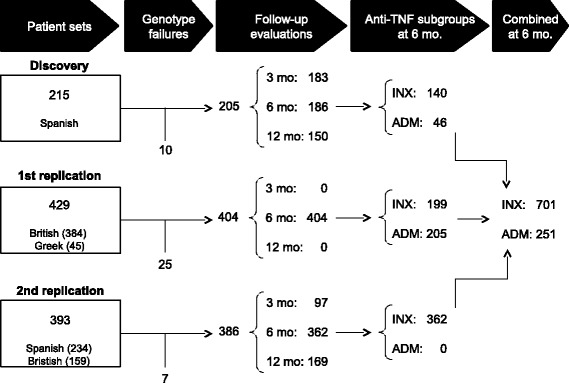
**Diagram representing the three rheumatoid arthritis patient sets and their composition.**

The three sets of patients with RA included in the study are shown in Figure 2. The discovery set included 215 patients of Spanish ancestry with RA who were recruited in six Spanish hospitals. Evaluations included the disease activity score in 28 joints (DAS28), which was available at the start of treatment with INX or ADM and after three, six and twelve months. The first replication set consisted of 429 patients treated with INX or ADM, 384 from the Biologics in Rheumatoid Arthritis Genetics and Genomics Study Syndicate (BRAGGSS), all of them with UK white ancestry, and 45 patients from two Greek hospitals, all of them of Greek ancestry (Figure 2). The DAS28 of these patients was evaluated at the start and after six months of treatment with INX or ADM. The second replication set of 393 patients with RA, all treated with INX, was subsequently collected in twelve Spanish hospitals (nine new hospitals plus new patients from three of the hospitals contributing to the discovery set, n = 234 Spanish with white ancestry) and by BRAGGSS (n = 159 UK whites). Analyses were limited to the patients with valid genotypes (95.3, 94.2 and 98.2% in the three patient sets, respectively): 205 from the discovery set (183 with three months follow-up, 186 with six months follow-up and 150 with twelve months follow-up); 404 from the first replication set (all with six month follow-up); and 386 from the second replication set (97 with three months of follow-up, 362 with six months of follow-up and 169 with twelve months of follow-up). All were biologic-naïve at treatment start. The indication of treatment, choice of drug and control of evolution were performed independently of this study. All patients provided their written informed consent. Collection of samples was approved by the local ethics committees (listed in the Appendix) and the study was approved by the Comité Ético de Investigación Clínica de Galicia (registry numbers 2009/173, 2011/162 and 2013/156).

### G1m allotype genotyping

The G1m allotypes were studied at the DNA level [17,18]. We have genotyped two nsSNPs encoding them: *rs1071803* (which A allele codes for the G1m17 allotype, whereas the G allele codes for the G1m3 allotype) and *rs11621259* (which C allele codes for one of the two amino-acids comprising the G1m1 allotype, whereas the A allele codes for a protein that does not induce antibodies, namely a null allotype). Due to paralog sequences in other *IGH* genes, there are no tests able to discriminate with confidence between G/A and A/A genotypes at *rs1071803* and between C/A and C/C genotypes at *rs11621259* (Figure 1). A carrier analysis was therefore performed that distinguished carriers and non-carriers of the G1m1,17 allotypes. Genotypes were obtained by PCR amplification followed by single-base extension with the SNaPshot Multiplex Kit (Applied Biosystems, Foster City, California). Samples with different genotypes were sequenced to assess the accuracy of results. The primers used are listed in Additional file 1: Table S1.

### Statistical analysis

The Statistica 7.0 (Statsoft) software was used throughout. Appropriate analyses were performed to evaluate differences in clinical characteristics stratified according to the specific anti-TNF (Table 1). Thus, 2 × 2 tables with Chi-square tests were used for comparing dichotomous clinical characteristics, 2 × 3 tables for variables with three levels as the EULAR response criteria, while t-tests were used to evaluate differences between quantitative clinical characteristics as ΔDAS28, baseline C-reactive protein (CRP) or baseline erythrocyte sedimentation rate (ESR). Response to treatment was considered as ΔDAS28 or according to the EULAR criteria [33]. A generalized linear model for ΔDAS28 and a logistic regression model for the EULAR criteria (confronting good responders with non-responders; moderate responders were not considered) were fitted. Unadjusted and adjusted analyses were conducted. Included covariates are indicated in each analysis. Samples with missing data for any of the variables implicated in each specific analysis were excluded. Meta-analysis of the beta coefficients corresponding to the regression models for the three sets of patients was done according to a fixed effects model using inverse-variance weights as implemented in the *meta* library of the R project [34].

**Table 1:** **Clinical characteristics of the patients with rheumatoid arthritis** ^**a**^ **from the discovery collection**

**Features**	**All patients**	**Infliximab**	**Adalimumab**
Patients, number (%)^a^	205	151 (73.7)	54 (26.3)
Female (%)	83.4	85.4	77.8
Age at diagnosis, median (IQR)	47 (38 to 55)	47 (37 to 55)	48 (40 to 56)
Diagnosis to anti-TNF, median (IQR)	6 (3 to 12)	6 (3 to 12)	6 (2 to 11)
RF, %	74.5	74.7	74.1
ACPA, %	75.7	75.4	76.9
Erosive arthritis, %	84.9	83.5	88.9
Smoking, %	13.9	11.8	20.0
DMARDs before anti-TNF, mean ± SD	2.5 ± 1.2	2.5 ± 1.2	2.6 ± 1.1
Concomitant DMARDs (%)	97.5	98.0	96.3
Baseline ESR, median (IQR)^b^	34 (19 to 54)	34 (19 to 53)	34 (19 to 57)
Baseline CRP (mg/L), median (IQR)^b^	11.5 (5.5 to 23.9)	11.5 (5.5 to 23.9)	14.3 (5.5 to 23)
Baseline HAQ, median (IQR)^b^	1.5 (1.0 to 2.0)	1.5 (1.0 to 2.0)	1.4 (1.0 to 2.0)
DAS28, (mean ± SD)			
baseline	5.9 ± 1.2	5.9 ± 1.1	5.9 ± 1.4
three months	3.9 ± 1.4	4.0 ± 1.5	3.5 ± 1.1
six months	3.8 ± 1.4	4.0 ± 1.5	3.4 ± 1.2
twelve months	3.7 ± 1.5	3.8 ± 1.5	3.4 ± 1.6
EULAR response, %			
three months			
responder	30.4	28.7	34.6
moderate	50.8	50.4	51.9
no-responder	18.8	20.9	13.5
six months			
responder	32.8	32.1	34.8
moderate	43.5	40.7	52.2
no-responder	23.7	27.1	13.0
twelve months			
responder	43.5	40.2	51.1
moderate	35.1	40.2	23.4
no-responder	21.4	19.6	25.5

^a^Only patients with successful genotypes are included; ^b^data from <85% of patients: 157 for baseline ESR, 126 for baseline CRP and 171 for baseline HAQ. ACPA = anti-cyclic citrullinated peptides; CRP = C-reactive protein; DAS28 = Disease Activity Score 28; DMARD = disease modifying anti-rheumatic drugs; ESR = erythrocyte sedimentation rate; EULAR = European League Against Rheumatism; HAQ = Health Assessment Questionnaire; IQR = interquartile range; RF = rheumatoid factor; SD = standard deviation.

## Results

### Association in the discovery patient set

Patients with RA in the discovery collection showed a median of six years between diagnosis and start of the treatment with INX or ADM (Table 1). They had clinical characteristics of severe disease: 85.3% were seropositive, 84.9% had developed joint erosions and they had an active disease (baseline DAS28 = 5.9 ± 1.2) after treatment with a mean of 2.5 disease-modifying antirheumatic drugs (DMARDs). The assessed treatment was with the first biologic administered and it was most often INX (73.7%). The patients treated with INX showed a lower decrease in DAS28 at three and six months than the patients treated with ADM, indicating the need to consider the drug as an important variable in subsequent analyses. There were no other significant differences between the patients treated with the two anti-TNF drugs.

Results of the two nsSNPs for the G1m1,17 allotypes were fully concordant confirming the perfect LD between them. Slightly less than half the patients (49.3%) did not carry the G1m1,17 allotypes and, therefore, were incompatible with the INX and ADM allotypes. A significant association was found in unadjusted analysis between lower response (ΔDAS28) and incompatibility at the G1m1,17 allotypes after six months of treatment (Table 2). This association remained significant after adjusting for gender, baseline DAS28, rheumatoid factor (RF) and anti-TNF (frequency data provided in Additional file 1: Table S2). Stratified analysis by anti-TNF showed significant association in the two strata, but only with INX in the adjusted analysis (Table 2). Association in patients treated with ADM was dubious because it disappeared after adjusting for the covariates, but the effect size was equal to that shown in patients treated with INX (Beta = 0.16). Therefore, the lack of association could be attributed to the lower number of patients treated with ADM than with INX and reduced statistical power (Table 2). No association between ΔDAS28 and G1m1,17 carrier status was found either at three or twelve months of treatment, but nominal differences were in the same direction as that observed at six months (data not shown). No significant association was detected using the EULAR response criteria at any time of follow-up (Additional file 1: Table S3).

**Table 2:** **Association of incompatibility at G1m (G1m1,17- genotype) with less improvement in DAS28 at six months of treatment with anti-TNF in the discovery samples**

					**Unadjusted**		**Adjusted** ^**b**^	**Adjusted** ^**b**^
	**G1m genotype** ^**a**^	**number**	**Baseline DAS28**	**ΔDAS28**	**Beta**	***P-value***	**Beta**	***P-value***
All patients	G1m1,17+	92	5.9 ± 1.1	2.3 ± 1.6	0.20	0.006	0.14	0.02
	G1m1,17-	94	5.7 ± 1.1	1.6 ± 1.5				
INX	G1m1,17+	68	5.9 ± 1.1	2.1 ± 1.6	0.16	0.06	0.16	0.03
	G1m1,17-	72	5.8 ± 1.1	1.6 ± 1.5				
ADM	G1m1,17+	24	6.1 ± 1.3	2.9 ± 1.5	0.32	0.03	0.16	0.19
	G1m1,17-	22	5.6 ± 1.4	1.9 ± 1.5				

^a^Carrier status; ^b^analyses adjusted for baseline DAS28, gender and RF (and anti-TNF for All patients). ADM, adalimumab; anti-TNF, anti-tumor necrosis factor; DAS28, disease activity score in 28 joints; INX, infliximab; RF, rheumatoid factor.

### First replication set

New patient samples from the UK and from Greece were obtained to replicate the previous findings. These patients showed some clinical features that were different from the discovery set of patients in baseline DAS28, baseline Health Assessment Questionnaire (HAQ), erosions, time since disease diagnosis and smoking (Tables 1 and 3). However, these patients also showed an established disease (median of 10 years from diagnosis to the start of the first anti-TNF treatment), showing high activity (baseline DAS28 = 6.5 ± 1.1), frequent erosions (58.5%) and seropositivity (79.7%). There were some differences between patients treated with INX and those treated with ADM in this replication set: the INX treated patients were younger at diagnosis, showed more joint erosions and responded with less decrease in DAS28 than the patients treated with ADM. Therefore, it was also necessary to consider the anti-TNF in the analysis.

**Table 3:** **Clinical characteristics of the patients with RA from the first and second replication collections**

	**First replication**			**Second replication**
**Features**	**All patients**	**Infliximab**	**Adalimumab**	**Infliximab**
Patients, number (%)	404	199 (49.3)	205 (50.7)	386
Female (%)	77.2	77.9	76.6	89.1
Age at diagnosis, median (IQR)	46 (34 to 53)	42 (33 to 51)	48 (36 to 56)	44 (35 to 51)
Diagnosis to anti-TNF, median (IQR)	10 (5 to 17)	11 (6 to 18)	9 (4 to 17)	8 (4 to 15)
ACPA, %^a^	77.4	79.4	75.5	60.9
Erosive arthritis, %^a^	58.2	65.0	51.1	77.6
Smoking, %^a^	56.6	56.8	56.3	26.1
Concomitant DMARDs (%)^a^	100	100	100	96.4
Baseline HAQ, median (IQR)	2.0 (1.6 to 2.4)	2.0 (1.8 to 2.5)	2.0 (1.5 to 2.3)	1.9 (1.4 to 2.3)
DAS28, (mean ± SD)				
baseline	6.5 ± 1.1	6.7 ± 1.0	6.3 ± 1.1	6.1 ± 1.2
six months	4.1 ± 1.3	4.3 ± 1.3	3.8 ± 1.2	4.1 ± 1.5
six months EULAR response, %				
responder	21.3	17.1	25.4	29.2
moderate	69.6	73.4	65.9	46.9
no-responder	9.2	9.5	8.8	24.0

^a^Data from <85% of patients: in the first replication (310 patients for concomitant DMARDs); in the second replication (196 patients for erosive arthritis; 174 for ACPA; 115 patients for smoking). ACPA, anti-citrullinated peptide antibodies; ADM, adalimumab; anti-TNF, anti-tumor necrosis factor; DAS28, disease activity score in 28 joints; DMARDs, disease modifying antirheumatic drugs; EULAR, European League against Rheumatism; HAQ, Health Assessment Questionnaire; INX, infliximab; IQR, interquartile range; RA, rheumatoid arthritis; SD, standard deviation.

More than half of the patients in this set (56.4%) were carriers of the G1m1,17 allotypes. Non-carrier status was associated with a lower response as evaluated with ΔDAS28, but only in the patients treated with INX (n = 199; Table 4). The association was significant, both in the unadjusted and in the adjusted analyses, replicating the finding of the discovery set. No association was found in the patients treated with ADM (Table 4). Analysis of the secondary outcome, EULAR response criteria, also showed significant association between incompatible allotypes and worse response to INX (*P* = 0.03; Additional file 1: Table S4), but not to ADM.

**Table 4:** **Association of G1m status with change in DAS28 at 6 months of treatment with anti-TNF**

					**Unadjusted**		**Adjusted** ^**b**^	
**First replication**	**G1m genotype** ^**a**^	**number**	**Baseline DAS28**	**ΔDAS28**	**Beta**	***P-value***	**Beta**	***P-value***
All patients	G1m1,17+	228	6.4 ± 1.1	2.5 ± 1.3	0.03	0.52	0.07	0.13
	G1m1,17-	176	6.6 ± 1.1	2.4 ± 1.3				
INX	G1m1,17+	116	6.7 ± 0.9	2.5 ± 1.2	0.14	0.04	0.15	0.02
	G1m1,17-	83	6.8 ± 1.1	2.2 ± 1.3				
ADM	G1m1,17+	112	6.1 ± 1.1	2.4 ± 1.3	−0.07	0.32	−0.01	0.83
	G1m1,17-	93	6.4 ± 1.0	2.8 ± 1.3				
**Second replication**								
INX	G1m1,17+	203	6.2 ± 1.1	2.2 ± 1.5	0.11	0.03	0.08	0.08
	G1m1,17-	159	6.1 ± 1.3	1.9 ± 1.5				

^a^Carrier status; ^b^analyses adjusted for baseline DAS28 and gender (and anti-TNF for All patients). ADM, adalimumab; DAS28, disease activity score in 28 joints; IFX, infliximab.

### Second replication set

Subsequently, a new set of patients with RA treated with INX was collected in Spanish hospitals and in the BRAGGSS. These patients also showed active disease with high baseline DAS28 (6.1 ± 1.2) and HAQ (1.9 (1.4 to 2.3)), a long evolution and common erosions (77.6%), but with a lower frequency of seropositivity than the two previous patient sets (Table 3). Response to treatment at six months was more similar to the discovery collection, with more balanced proportions of the three response classes, than to the first replication set.

Patients in this second replication set also showed an association between lower response to treatment with INX at six months and incompatibility at the G1m1,17 allotype (Table 4, lower rows), but the difference was only significant before adjusting for covariates (*P* = 0.03, *versus P* = 0.08 after adjustment by baseline DAS28 and gender). Comparison of responder with non-responder patients according to the EULAR criteria did not show significant differences (Additional file 1: Table S4).

### Combined analysis

Results from the patients with RA in the three previous sets were combined by simple pooling and by meta-analysis. Pooled analysis showed a significantly worse response at six months in the patients treated with INX who were carriers of the incompatible allotypes (Table 5). Very similar results were obtained by meta-analysis: significant association between higher ΔDAS28 and G1m1,17 compatibility (Beta = 0.12, 95% confidence interval (C.I.) = 0.05 to 0.19, *P* = 0.0005) with no heterogeneity between the three patient sets (I^2^ = 0%, 95% C.I. = 0.0 to 81.4%). In addition, analysis according to the EULAR criteria showed that the compatible patients were more commonly responders and less commonly non-responders to INX than the incompatible patients (30.1% *versus* 21.5% responders and 16.9% *versus* 22.4% non-responders; *P* = 0.03). On the contrary, combined analysis of response to ADM at six months (Table 5), or to any of the two anti-TNF at three or twelve months (not shown) did not show significant differences between G1m1,17 carriers and non-carriers.

**Table 5:** **Combined analysis of G1m status according to change in DAS28 and to the EULAR criteria**

**Strata**	**G1m genotype** ^**a**^	**number**	**Baseline DAS28**	**ΔDAS28**	**Beta** ^**b**^	***P-value*** ^**b**^	**R** ^**c**^	**NR** ^**c**^	**OR** ^**b**^	***P-value*** ^**b**^
All patients	G1m1,17+	523	6.3 ± 1.1	2.3 ± 1.4	0.09	0.002	153 (29.3)	79 (15.1)	1.5	0.035
	G1m1,17-	429	6.2 ± 1.2	2.0 ± 1.5			98 (22.8)	90 (21.0)		
INX	G1m1,17+	387	6.3 ± 1.1	2.3 ± 1.5	0.11	0.001	116 (30.0)	67 (17.3)	1.5	0.03
	G1m1,17-	314	6.2 ± 1.3	1.9 ± 1.5			67 (21.3)	78 (24.8)		
ADM	G1m1,17+	136	6.2 ± 1.1	2.5 ± 1.4	0.03	0.55	37 (27.2)	12 (8.8)	1.1	0.57
	G1m1,17-	115	6.2 ± 1.2	2.5 ± 1.4			31 (27.0)	12 (10.4)		

^a^Carrier status; ^b^analyses adjusted for baseline DAS28 and gender (and anti-TNF for All patients); ^c^number (% response class/all patients with this genotype). ADM, adalimumab; DAS28, disease activity score in 28 joints; IFX, infliximab; NR, non-responders; R, responders.

Analysis of ΔDAS28 in function of the G1m1,17 status was repeated adjusting for all characteristics that were different between the discovery and replication sets (baseline DAS28, gender, baseline HAQ, age at diagnosis, years from diagnosis to treatment, erosive arthritis, anti-citrullinated peptide antibodies (ACPA) and smoking; Additional file 1: Table S5). Results were very similar to those already shown in spite of the exclusion of patients lacking data for any of the new covariates (Beta = 0.13, *P* = 0.01 for the 304 patients treated with INX who had complete information). We also checked the effect of stratification by the clinical characteristics taken individually. There was a strong effect of the ACPA status. A stronger association was observed in the ACPA negative patients receiving INX (n = 132, Beta = 0.26, *P* = 0.00092) than in the whole set of patients, whereas it was reduced and non-significant in the ACPA positive subgroup (n = 342, Beta = 0.06, *P* = 0.19). In addition, stratification by ACPA status of the patients receiving INX also showed association at 12 months of follow-up in the ACPA negative patients (n = 81, Beta = 0.22, *P* = 0.022 *versus* n = 164, Beta = −0.08, *P* = 0.3 in the ACPA positive patients). No effect was found at three months. Also, the association was stronger in the patients who initiated treatment at a younger age than the median of the whole set of patients (56 years) than in the group that started treatment older than 56 years (Beta = 0.20, *P* = 0.000040 in the 369 patients starting INX at ≤56 years *versus* Beta = −0.005, *P* = 0.9 in the 313 older patients) and in those in whom treatment was delayed less since diagnosis of RA (Beta = 0.20, *P* = 0.000054 in the 315 patients treated with INX ≤8 years after diagnosis, *versus* Beta = 0.02, *P* = 0.7 in the 359 with >8 years of delay). These two variables, age or treatment initiation and delay since diagnosis were weakly correlated (r = 0.22), and, consequently, the effect of one was not explained by the effect of the other. In addition, we checked the effect of considering the concomitant use of methotrexate, which was the DMARD used by 85.1% of the 883 patients with this information. The results changed very little, both when considering all the patients, or separately the patients treated with INX (n = 661, Beta = 0.11, *P* = 0.0016). The association showed a trend to be stronger in the patients taking methotrexate (Beta = 0.12), but the low number of patients in the group not taking this DMARD made the results inconclusive. Similarly, no differences in the effect of the Gm1,17 allotype were detected with the other clinical variables analyzed (smoking habit, RF status and age at diagnosis).

## Discussion

Our results showed a consistent association between the G1m allotypes and response to treatment with INX at six months of follow-up. A decreased ΔDAS28 was observed in the patients with incompatible allotypes in the discovery patients and in the two replication sets. In addition, fewer responders and more non-responders were present among the G1m1,17 incompatible patients than among the compatible patients. The effect of G1m incompatibility was weak in the whole set of patients, but stronger in some patient subgroups. Therefore, we think that it could be of clinical utility if it is confirmed in further studies.

We consider the association of response to INX as consistently reproduced in spite of the *P* = 0.08 obtained in the second replication, which is over the pre-specified significance threshold. This opinion is supported by two arguments. First, this study hypothesized a lower response in the incompatible patients, which is the result that has been found. Therefore, single-tailed tests could have been used in place of two-tailed tests [35]. Single-tailed tests would lead to more significant differences in the three patient sets including *P* = 0.04 in the second replication. Second, some authors have recommended applying a lower significance threshold for replication than for discovery to compensate for the overestimated effect sizes commonly obtained in the first study showing significant association [36,37]. In any case, confirmation by independent researchers is needed for validation of this biomarker. Unfortunately, we cannot find support in the published GWAS of response to anti-TNF because they did not include the allotype-specific nsSNPs and showed low coverage of the *IGH* locus [23,28-32].

The requirement of replication is still more important for the subgroup analyses that we only report here as hypothesis generating exploratory results. This caution is necessary because subgroup analyses are especially prone to false positive results [38]. Therefore, the stronger associations found in ACPA negative patients than in ACPA positive patients, in patients younger than the median age at treatment initiation and in those patients with a short delay in treatment since diagnosis should be taken with caution. These results, if confirmed, could reflect the differences in immune responsiveness between the subgroups of patients.

In effect, our results are concordant with the initial hypothesis involving induction of immune responses against the incompatible allotype of INX. These immune responses could include anti-allotype antibodies [16,17,19] or a T cell response independent of anti-allotype antibodies [18]. The anti-allotype antibodies would target the INX molecule leading to its accelerated clearance. Anti-allotype antibodies have been found in polytransfused subjects and in multiparous women [16] who were exposed to blood containing allotype incompatible immunoglobulins. However, these antibodies have not been found in some studies that have searched for them after administration of IgG1-based drugs [17,20,39]. These negative results have been attributed to the technical difficulty of the assays [16,17,19,22,39]. In this respect, the authors who were successful in finding anti-allotype antibodies in patients treated with IgG1-based drugs had used very sensitive assays, which included few washes to avoid dissociation of low affinity antibodies and acid dissociation of pre-existing immunocomplexes [19]. Our approach, at the DNA level instead of the anti-allotype antibodies, has avoided these technical difficulties, has permitted us to use patients lacking sera at the relevant point in the treatment course, and allows for other forms of involvement of the allotypes beyond the induction of antibodies. One of these alternative mechanisms is a different type of immune response against the G1m1 allotype that involves CD4 T cell activation and production of cytokines [18]. The T cell antigen does not include the allotype but a nearby peptide that becomes accessible to antigen processing and presentation because the G1m1 allotype introduces an asparaginyl endopeptidase cleavage site [18]. None of the two alternatives, antibodies or T cell responses, could be tested in our patients because of unavailability of the relevant samples.

Alternative mechanisms for the association that do not involve anti-drug immune reactions could also be invoked. They are suggested by differences in the strength of antibody responses against autoantigens, pathogens and cancer antigens associated with the G1m allotypes [20,23-25]. These associations could be due to the allotypes themselves, because they modify the interaction with Fc-gamma receptors [40], or to other variants in the immunoglobulin heavy chain locus that is very polymorphic, displays extensive LD and includes the sequences of all immunoglobulin heavy chains [26,27].

The associations were observed in the three sets of patients at six months with ΔDAS28, but only in the first replication and in the combined analysis with the EULAR response. This discordance between the outcomes is congruent with the lower sensitivity to change of the EULAR response, which was considered as a dichotomous variable, than of ΔDAS28, which is a continuous variable [41,42]. Another aspect of our study deserving comment is that results at three or twelve months of follow-up were non-significant (although the same direction of change was observed). These negative results could be ascribed to less powerful analysis due to fewer patients with data available at these times than at six months (Figure 2), or to latency in the development of an immune response preventing association at three months, or to change of treatment in patients showing poor response at six months. In an attempt to compensate by the latter confounding factor, we included the 33 patients that we knew had interrupted treatment after six months due to inefficacy in the EULAR response analysis at twelve months (as non-responders), but this analysis did not show association (not shown). In addition, it is possible that differences in the composition of the patient sets at the different times of evaluation contribute to the discrepant results. This alternative is suggested by the demonstration of association at twelve months of follow-up in the subset of ACPA negative patients, which was also the subset with the strongest association at six months of follow-up. At present, we cannot discriminate between the discussed possibilities.

Differences between the discovery and the replication sets included origin within Europe, baseline disease activity, time of evolution, seropositivity, erosions and smoking. Reproducibility of the association across these differences suggests that it is robust and that could be confirmed in additional studies. This replication is a requirement before defining the applicability of our findings. However, we can already comment on the effect size of the INX association we have observed. The mean difference in ΔDAS28 (which is larger than the attributable effect according to linear regression analysis probably because it includes effects attributable to other covariates) between G1m1,17 compatible and incompatible patients after six months in INX was 0.4. This is below 0.6, the change considered significant in the EULAR criteria [33] but similar to the difference in ΔDAS28 between seropositive and seronegative RA patients treated with rituximab at six months (0.3 for RF and 0.6 for ACPA according to a meta-analysis of patient registers [5]; and 0.35 for seropositive patients from a meta-analysis of clinical trials [6]), which is considered sufficient to help in the choice of treatment. Therefore, it is possible that the G1m1,17 allotypes could become useful, after further confirmation and analysis, to inform drug choice in RA treatment. This could be especially true in some subgroups of patients as suggested by the strong association in ACPA negative patients and in young patients and those initiating treatment early.

In contrast with INX, patients treated with ADM did not show consistent association between response and G1m1,17 compatibility. The total number of ADM treated patients was not enough to exclude with sufficient power an effect as was observed with INX (643 patients will be needed to exclude with 0.8 power an effect size of slope = 0.11). However, it is possible that G1m incompatibility does not affect response to ADM because there is already precedent of a study showing that response of RA patients to ADM is not affected by their allotype [20]. In addition, several differences between INX and ADM make more likely the development of immune responses against the first. They include the larger immunogenicity of other parts of the INX molecule, which is a mouse/human chimera, than of ADM, which is a human antibody; the different routes of administration and much wider fluctuations of INX than of ADM, and the development of neutralizing anti-idiotypic antibodies against ADM that could reflect the immunodominance of the ADM paratope over other epitopes [43]. The large fluctuations of INX concentration are due to its intravenous administration every eight weeks in contrast with the subcutaneous administration every two weeks of ADM [44].

Other biologics used for the treatment of RA bear the G1m1 or G1m17 allotypes and it will be interesting to study if the response to them is affected by the allotype of the patients. Rituximab and tocilizumab bear the G1m1,17 allotypes, whereas abatacept has the G1m1 allotype and certolizumab pegol the G1m17 allotype due to the incomplete presence of the IgG1 constant region in their molecules [17,45-47]. Etanercept, in turn, cannot induce any anti-allotype responses because it lacks the G1m3/G1m17 allotypes and bears the null allotype at G1m1 [46]. We were unable to find information about the allotypes of golimumab (although, it is described as bearing the INX IgG1 heavy chain [48]).

## Conclusions

The G1m1,17 allotypes of patients with RA have shown an association with response to INX treatment at six months of follow-up. This association was replicated in independent patient sets. The patients who were incompatible with the allotypes in the INX molecule showed a poorer response than the compatible ones. This pattern of response is consistent with the development of immune reactivity against INX. The difference in response between allotype compatible and incompatible patients was small, but of possible utility because it was of similar magnitude to the difference in response to rituximab between seronegative and seropositive patients, and because it was larger in some subgroups of patients.

## Appendix

The following ethics committees approved sample collection and this study: Comité Ético de Investigación Clínica de Galicia (currently, Comité Autnómico de Ética de la Investigacion de Galicia), Comité Ético de Investigación Clínica del Hospital Universitario Virgen Macarena, Comité de Ética de la Investigación Clínica Fundación de Gestión Sanitaria del Hospital de la Santa Creu i Sant Pau, University of Thessally and Medical School Ethics Committee, Comité Ético de Investigación del Hospital Carlos Haya de Málaga, Comité Ético de Investigación Clínica de la Ciutat Sanitária i Universitária de Bellvitge, Comité Ético de Investigación Clínica del Hospital Clínic i Provincial de Barcelona, Comité de Bioética del Consejo Superior de Investigaciones Científicas, Cómite de Ética de Investigación Clínica del Hospital Universitario de la Paz, Comité Ético de Investigación Clínica del Hospital 12 de Octubre, Comité Ético de Investigación Clínica del Hospital Clínico Universitario Virgen de la Arrixaca, Comité Ético de Investigación Clínica del Hospital Universitario Virgen de las Nieves, Comité Ético de Investigación Cclínica del Hospital Virgen Victoria de Málaga, Cómite Ético de Investigación Clínica del Hospital San Cecilio, Cómite Ético de Investigacion Clínica del Instituto de Investigación Sanitaria Gregorio Marañón, Comité Ético de Investigación Clínica del Hospital Universitario Doctor Pesset, UK Central Office of Research Ethics Committee.

## Additional file

Additional file 1: Table S1. Primers and probes in 5′ → 3′ direction employed in this study. All primers were selected with Primer3 and Oligos software. They were checked to avoid formation of dimers between the oligonucleotides included in the reaction. **Table S2.** Clinical characteristics of patients from the discovery set of patients stratified by treatment and G1m1,17 allotype. **Table S3.** Association of G1m carrier status with treatment response according to the EULAR criteria at six months of treatment with anti-TNF in the discovery samples. **Table S4.** Association of the G1m carrier status with treatment response according to the EULAR criteria at six months of treatment with anti-TNF in the replication collections. **Table S5.** Comparison between the clinical characteristics of the three RA patient sets (discovery and two replications) with six months follow-up for infliximab treatment.

## Abbreviations

ACPA: anti-citrullinated peptide antibodies
ADM: adalimumab
BRAGGSS: Biologics in Rheumatoid Arthritis Genetics and Genomics Study Syndicate
C.I: confidence interval
CRP: C-reactive protein
DAS28: disease activity score in 28 joints
DMARDs: disease-modifying antirheumatic drugs
ESR: erythrocyte sedimentation rate
EULAR: European League against Rheumatism
Fc: fragment crystallizable
G1m: genetic marker in constant region of immunoglobulin gamma heavy chain of subclass1
GWAS: genome-wide association study
HAQ: Health Assessment Questionnaire
IgG: immunoglobulin G
IgG1: immunoglobulin G subclass 1
IGH: immunoglobulin heavy chain
IGHG: immunoglobulin heavy constant gamma
INX: infliximab
IQR: interquartile range
LD: linkage disequilibrium
nsSNP: non synonymous single nucleotide polymorphism
PCR: polymerase chain reaction
RA: rheumatoid arthritis
RF: rheumatoid factor
SD: standard deviation
SNP: single nucleotide polymorphism
TNF: tumor necrosis factor
