# Supplementary material for: Rheumatoid arthritis response to treatment across IgG1 allotype – anti-TNF incompatibility: a case-only study
Source: Arthritis Res Ther. 2015 Mar 18;17(1):63. doi: 10.1186/s13075-015-0571-z (PMC4411723; doi:10.1186/s13075-015-0571-z)
Supplement: Additional file 1: Table S1. — Primers and probes in 5′ → 3′ direction employed in this study. All primers were selected with Primer3 and Oligos software. They were checked to avoid formation of dimers between the oligonucleotides included in the reaction. Table S2. Clinical characteristics of patients from the discovery set of patients stratified by treatment and G1m1,17 allotype. Table S3. Association of G1m carrier status with treatment response according to the EULAR criteria at six months of treatment with anti-TNF in the discovery samples. Table S4. Association of the G1m carrier status with treatment response according to the EULAR criteria at six months of treatment with anti-TNF in the replication collections. Table S5. Comparison between the clinical characteristics of the three RA patient sets (discovery and two replications) with six months follow-up for infliximab treatment. [file 13075_2015_571_MOESM1_ESM.doc]

**Table S1**. Primers and probes in 5’→ 3’ direction employed in this study. All primers were selected with Primer3 and Oligos software. They were checked to avoid formation of dimers between the oligonucleotides included in the reaction.

|  |  | |  |
| --- | --- | --- | --- |
| SNP | Allotype Forward Primer | Reverse Primer | Minisequencing Probe |
| **rs 1071803** | G1m3 / G1M17 GGGCACCCAGACCTACATC | AGAAGACCCTCTCCCTGAGC | tcacaagcccagcaacaccaaggtggacaaga |
| **rs11621259** | G1m1 CGGCCCACCCTCTGCCCTGA | GCTGTAGAGGAAGAAGGAGCCGTC | gtcaggctgaca*tggttcttggtca |
|  |  |  |  |
| **Primers for sequencing** |  |  |  |
| **G1m3/17 R** | AGGCTGAAGGGAACGTGAG |  |  |
| **G1m1 R** | GCGATGTCGCTGGGATAGAA |  |  |

*This nucleotide has been modified (A in place of C) to avoid self-priming

The multiplex SNaPshot reaction was performed in a final volume of 10

μl, containing one-fifth of the PCR reaction, 2.5

μl of the SNaPshot Multiplex Ready Reaction Mix, 1

μl of sequencing buffer from the Big Dye V3.1 Terminator Kit and SNaPshot primers at a concentration of 0.02–0.05

μM. Cycling conditions were 25 cycles of rapid thermal ramp to 96°C, 96°C for 10

s; rapid thermal ramp to 50°C, 50°C for 5

s; and rapid thermal ramp to 60°C and 60°C for 30

s. SNaPshot products were then treated 1

h at 37°C with 3

U of shrimp alkaline phosphatase (Amersham Biosciences/GE Healthcare Europe GmbH, Saclay, France). After heat inactivation of the alkaline phosphatase 15

min at 75°C, labelled products were separated using a 25

min run on an ABI Prism 3130xL DNA sequencer and data were analysed using the GeneMapper Analysis Software version 4.0 (Applied Biosystems).

**Table S2**. Clinical characteristics of patients from the discovery set of patients stratified by treatment and G1m1,17 allotype

|  | All patients (205) | | Infliximab (151) | | Adalimumab (54) | |
| --- | --- | --- | --- | --- | --- | --- |
|  | G1m1,17- | G1m1,17+ | G1m1,17- | G1m1,17+ | G1m1,17- | G1m1,17+ |
| Patients, number (%)^a^ | 101 (49.3) | 104 (50.7) | 73 (48.3) | 78 (51.7) | 28 (51.9) | 26 (48.1) |
| Female (%) | 85.1 | 81.7 | 90.4 | 80.8 | 71.4 | 84.6 |
| Age at diagnosis, median (IQR) | 46 (37-54) | 48 (39-56) | 47 (37-55) | 48 (39-55) | 46(38-52) | 48(41-59) |
| Diagnosis to anti-TNF, median (IQR) | 6 (2-12) | 6 (3-12) | 6 (3-12) | 6(3-13) | 6 (2-10) | 7 (3-11) |
| RF, % | 75.2 | 73.8 | 72.6 | 76.6 | 82.1 | 65.4 |
| Anti-CCP, % | 70.6 | 80.4 | 69.2 | 80.8 | 75.0 | 78.9 |
| Erosive arthritis, % | 85.7 | 84.2 | 84.3 | 82.7 | 89.3 | 88.5 |
| Smoking, % | 15.6 | 12.2 | 11.3 | 12.3 | 28.0 | 12.0 |
| DMARDs before of anti-TNF, mean ± SD | 2.5 ± 1.2 | 2.6 ± 1.3 | 2.4 ± 1.1 | 2.6 ± 1.4 | 2.6 ± 1.1 | 2.7± 1.2 |
| Concomitant DMARDs (%)**^c^** | 85.1 | 81.7 | 98.6 | 97.4 | 92.9 | 100.0 |
| Baseline ESR, median (IQR)**^c^** | 39.5 (19-56) | 32 (19-52) | 35 (16-53) | 32 (20-54) | 47 (23-70) | 33 (18-46) |
| Baseline CRP (mg/L), median (IQR)**^c^** | 11.7 (5.2-25.4) | 11.6(5.8-21.7) | 10.1 (5.0-23.2) | 11.6 (5.9-32.5) | 21 (6-67) | 10.3 (4.5-20.5) |
| Baseline HAQ, median (IQR)^c^ | 1.4 (1-1.88) | 1.5 (1.1-2.3) | 1.4 (0.9-1.9) | 1.6 (1.3-2.3) | 1.6 (1.0-1.9) | 1.3 (0.9-2.1) |
| DAS28, (mean ± SD) |  |  |  |  |  |  |
| baseline | 5.7 ± 1.2 | 6.0 ± 1.2 | 5.8 ± 1.1 | 5.9 ± 1.1 | 5.6 ± 1.4 | 6.1 ± 1.4 |
| 3 months | 3.7 ± 1.3 | 3.7 ± 1.5 | 4.0 ± 1.4 | 4.0 ± 1.6 | 3.4 ± 1.1 | 3.6 ± 1.2 |
| 6 months | 4.0 ± 1.4 | 3.4± 1.5 | 3.8 ± 1.6 | 4.2 ± 1.5 | 3.6 ± 1.2 | 3.2 ± 1.1 |
| 12 months | 3.7 ± 1.5 | 3.3 ± 1.5 | 3.6 ± 1.4 | 3.9 ± 1.6 | 3.1 ± 1.4 | 3.8 ± 1.8 |
| EULAR response, % |  |  |  |  |  |  |
| 3 months |  |  |  |  |  |  |
| responder | 29.3 | 31.5 | 26.2 | 31.3 | 27.3 | 41.7 |
| moderate | 51.1 | 50.6 | 53.8 | 46.9 | 50.0 | 54.2 |
| no-responder | 19.6 | 18.0 | 20.0 | 21.9 | 22.7 | 4.2 |
| 6 months |  |  |  |  |  |  |
| responder | 27.7 | 38.0 | 27.8 | 36.8 | 37.0 | 32.0 |
| moderate | 43.6 | 43.5 | 41.7 | 39.7 | 44.4 | 60.0 |
| no-responder | 28.7 | 18.5 | 30.6 | 23.5 | 18.5 | 8.0 |
| 12 months |  |  |  |  |  |  |
| responder | 43.0 | 44.0 | 35.7 | 45.1 | 60.9 | 41.7 |
| moderate | 34.2 | 36.0 | 41.1 | 39.2 | 17.4 | 29.2 |
| no-responder | 22.8 | 20.0 | 23.2 | 15.7 | 21.7 | 29.2 |
|  |  |  |  |  |  |  |
|  |  |  |  |  |  |  |
|  |  |  |  |  |  |  |

**Table S3. Association of G1m carrier status with treatment response according to the EULAR criteria at 6 months of treatment with anti-TNF in the discovery samples**

|  | G1m genotype ^a^ | R^b^ | NR^b^ | OR^c^ | *P-value* |
| --- | --- | --- | --- | --- | --- |
| All | G1m1,17+ | 35 (38.0) | 17 (18.5) | 1.9 | 0.16 |
|  | G1m1,17- | 26 (27.7) | 27 (28.7) |  |  |
| INX |  |  |  |  |  |
|  | G1m1,17+ | 25 (36.8) | 16 (23.5) | 1.8 | 0.25 |
|  | G1m1,17- | 20 (27.8) | 22 (30.6) |  |  |
| ADM |  |  |  |  |  |
|  | G1m1,17+ | 10 (41.7) | 1 (4.2) | 4.1 | 0.32 |
|  | G1m1,17- | 6 (27.2) | 5 (22.7) |  |  |

1. Carrier status
2. Number (% response class/all patients with this genotype)
3. Analyses adjusted for baseline DAS28, gender and RF (and anti-TNF for All)

**Table S4. Association of the G1m carrier status with treatment response according to the EULAR criteria at 6 months of treatment with anti-TNF in the replication collections.**

|  | G1m genotype ^a^ | R^b^ | NR^b^ | OR^c^ | *P-value* | R^b^ | NR^b^ | OR^c^ | *P-value* |
| --- | --- | --- | --- | --- | --- | --- | --- | --- | --- |
| All | G1m1,17+ | 53 (23.2) | 20 (8.8) | 1.4 | 0.43 |  |  |  |  |
|  | G1m1,17- | 33 (18.8) | 17 (9.7) |  |  |  |  |  |  |
| INX |  |  |  |  |  |  |  |  |  |
|  | G1m1,17+ | 26 (22.4) | 9 (7.8) | 4.1 | 0.03 | 65 (32.0) | 42 (20.7) | 1.5 | 0.12 |
|  | G1m1,17- | 8 (9.6) | 10 (12.0) |  |  | 39 (24.5) | 46 (28.9) |  |  |
| ADM |  |  |  |  |  |  |  |  |  |
|  | G1m1,17+ | 27 (24.1) | 11 (9.8) | 0.69 | 0.51 |  |  |  |  |
|  | G1m1,17- | 25 (26.9) | 7 (7.5) |  |  |  |  |  |  |

^a^ Carrier status

^b^ R = responders, NR = non-responder. Values are number (and % response class/all patients with this genotype)

^c^ Analyses adjusted for baseline DAS28 and gender (and anti-TNF for All)

**Table S5.** Comparison between the clinical characteristics of the three RA patient sets (discovery and two replications) with 6 months follow-up for Infliximab treatment.

|  | Discovery |  | First  replication |  | Second replication | *P-value* |
| --- | --- | --- | --- | --- | --- | --- |
| Patients, number ^a^ | 140 |  | 199 |  | 362 |  |
| Female (%) | 85.4 |  | 77.9 |  | 89.8 | 0.002 |
| Age at diagnosis, median (IQR) | 47 (37-55) |  | 42 (33-51) |  | 44 (34-52) | 0.04 |
| Diagnosis to anti-TNF, median (IQR) | 6 (3-12) |  | 11 (6-18) |  | 8 (4-15) | 0.002 |
| ACPA, % ^b^ | 75.8 |  | 79.4 |  | 63.1 | 0.0005 |
| Erosive arthritis, % ^b^ | 83.5 |  | 65.0 |  | 77.1 | 0.0006 |
| Smoking, % ^b^ | 12.0 |  | 56.8 |  | 26.4 | 1 x 10^-16^ |
| Concomitant DMARDs (%)^b^ | 97.5 |  | 100 |  | 96.5 | 0.06 |
| Baseline HAQ, median (IQR) | 1.5 (1.0 - 2.0) |  | 2.0 (1.8- 2.5) |  | 1.9 (1.4-2.3) | 3.2 x 10^-7^ |
| DAS28, (mean ± SD) |  |  |  |  |  |  |
| baseline | 5.8 ± 1.1 |  | 6.7 ± 1.0 |  | 6.2 ± 1.2 | 5 x 10^-12^ |
| 6 months | 4.0 ± 1.5 |  | 4.3 ± 1.3 |  | 4.1 ± 1.5 | 0.05 |
| 6 mo. EULAR response, % |  |  |  |  |  |  |
| responder | 32.1 |  | 17.1 |  | 28.7 | 1.3 x 10^-7^ |
| moderate | 40.7 |  | 73.4 |  | 47.0 |  |
| no-responder | 27.1 |  | 9.5 |  | 24.3 |  |

1. Only patients with successful genotypes.
2. Data from < 85% of patients: discovery (108 for concomitant DMARDs), first replication (165 for ACPA), second replication (149 for ACPA, 170 for erosive arthritis, 106 for smoking and 303 for baseline HAQ )
